# Association between registered nurse staffing levels and in-hospital mortality in craniotomy patients using Korean National Health Insurance data

**DOI:** 10.1186/s12912-020-00430-0

**Published:** 2020-05-07

**Authors:** Yunmi Kim, Se Young Kim, Kyounga Lee

**Affiliations:** 1 College of Nursing, Eulji University, 553, Sanseong-daero, Sujeong-gu, Seongnam-si, Gyeonggi-do Republic of Korea; 2 Department of Nursing, Changwon National University, 20 Changwondaehak-ro, Uichang-gu, Changwon-si, Gyeongsangnam-do Republic of Korea; 3 Medical Research Collaborating Center, Seoul National University Hospital, 101 Daehak-ro, Jongno-gu, Seoul, Republic of Korea

**Keywords:** Registered nurse staffing level, In-hospital mortality, Craniotomy, Intensive care unit

## Abstract

**Background:**

The level of registered nurse (RN) staffing is a fundamental factor influencing patient safety. Craniotomy patients need intensive care after surgery, the majority of which is provided by RNs.

**Methods:**

This study was conducted to investigate the relationship of the RN staffing level of general wards and intensive care units (ICUs) with in-hospital mortality after craniotomy using Korean National Health Insurance claim data. The RN staffing level was categorized based on the bed-to-RN ratio.

**Results:**

The in-hospital mortality rate of craniotomy patients was elevated at hospitals with a high bed-to-RN ratio in general wards, ICUs, and hospitals overall. It was determined that in-hospital mortality of craniotomy patients could be decreased by more than 50% by reducing the bed-to-RN ratio from 4.5 or more to less than 3.5 in general wards, from 1.25 or more to less than 0.88 in ICUs, and from 2.5 or more to less than 1.67 in hospitals overall.

**Conclusions:**

Since the RN staffing level is related to the in-hospital mortality rate of craniotomy patients, a sufficient staffing level of RNs should be ensured to reduce the mortality of craniotomy patients.

## Background

Registered nurses (RNs) play a very important role in ensuring patient safety by working within a 24-h surveillance system in which they can promptly detect changes in the patient’s condition and provide immediately needed interventions [1]. Patient care is delivered by a team comprising a variety of personnel. When the provision of care by RNs in collaboration with other medical staff and assistants is below the needed level, patient safety is compromised and the incidence of adverse events increases. In particular, adequate RN staffing is essential for providing continuous patient care with effective surveillance because RNs have to monitor and manage their patients’ care needs [2]. Therefore, the level of RN staffing is a fundamental factor influencing patient safety [3].

In Korea, according to Article 38 of the Enforcement Rule of the Medical Service Act, hospitals are required to have one RN per 2.5 inpatients per day per year. However, more than 50% of hospitals do not satisfy this rule [4, 5]. Since punishment for violation of this rule is weak, it is virtually impossible to regulate hospitals that fail to comply. Therefore, the rule regarding the number of RNs is often considered to be a recommendation, not a mandatory requirement. In addition, since this rule does not vary across types of hospitals or departments, it is difficult to apply in practice. Since 1999, the Korean government has introduced a fee incentive policy as another way to improve RN staffing levels. This policy attempts to raise RN staffing levels by paying higher hospital admission fees to hospitals with higher RN staffing levels, and the RN staffing level is suggested as a grade based on the bed-to-RN ratio according to the type of hospital (tertiary hospitals or secondary hospitals) and department (general ward or intensive care unit [ICU]). The RN staffing levels of hospitals are disclosed to the public, so that patients and RNs can consider this factor when choosing a hospital for treatment or employment.

Hospitals with lower levels of RN staffing had higher patient mortality rates during hospitalization or within 30 days after admission [1, 3, 6–11]. Improving RN staffing reduced the inpatient mortality rate by 4.9 fewer deaths per 1000 elective cardiac surgery patients [3]. In a previous Korean study, the mortality of surgical patients during hospitalization was lowest at hospitals with the highest RN staffing level, and was higher at hospitals with lower levels of RN staffing [11]. A systematic review of 43 studies and an analysis of whether nurse staffing levels were related to patient, nurse employee, or hospital outcomes revealed that RN staffing was associated with the failure-to-rescue rate, inpatient mortality rate, and length of hospital stay [12]. However, the relationship between RN staffing levels and patient mortality rate is not always consistent, which is why further studies are needed. In a study investigating the relationship between nurse staffing levels and quality of care using data from 799 hospitals in the United States, RN staffing levels (as estimated in hours) were associated with better care for hospitalized patients. Higher levels of RN staffing were associated with lower rates of these adverse outcomes, whereas the staffing levels of licensed practical nurses or aides were not associated with lower rates of these outcomes [13]. Another study suggested that RN staffing levels and in-hospital mortality were related in secondary hospital ICUs, but not in tertiary hospital ICUs [9]. Every additional patient per full-time-equivalent RN was associated with a 9% increase in the odds of death in the ICUs of secondary hospitals.

One of the most important times during hospital admission is the postoperative period. Neurosurgical patients require high levels of nursing and vigilance and additional postoperative monitoring in the ICUs [14]. Craniotomy patients are among those with the most severe diseases, and are often in poor condition. During the week after surgery, these patients should receive frequent nursing care that includes thorough observations, as it may be difficult to identify postoperative complications in these patients [15]. In Korea, craniotomy patients have the highest mortality rate after surgery, and are also known to have the largest variation in mortality rate between hospitals [16]. Patients who undergo craniotomy are at a high risk of infection due to the insertion of various invasive instruments and/or the administration of immunosuppressant drugs for therapeutic purposes. To prevent post-craniotomy infections, it is necessary for RNs to closely monitor and assess the site of insertion of the ventricular drainage tube and to manage the drainage system using aseptic techniques [17].

Postoperative monitoring in an ICU environment after craniotomy is routine at many hospitals [18], in which ICU RNs play an important role. Stabilizing the patient’s physiological parameters and neurological state is a key priority, and successfully doing so ultimately affects patient mortality and outcomes [19]. Most patients who undergo craniotomy are treated in the ICU and transferred to the general ward after their condition is confirmed to be stable. Recently, however, as the question of the necessity of ICU care for craniotomy patients has been raised, some studies have reported that not all craniotomy patients need ICU care [18, 20]. This suggests that RNs in general wards, as well as in the ICUs, can now play an especially important role and have a significant impact on craniotomy patient outcomes. Thus, when considering the factors that influence the mortality rate in craniotomy patients, both general ward and ICU RN staffing levels should be considered. In addition, it has been suggested that more research should investigate a wider range of hospitals to examine the effects of RN staffing on patient mortality [10]. Therefore, to overcome the limitations of previous studies, we analyzed RN staffing levels in general wards, ICUs, and hospitals overall using nationwide big data to clarify the relationship between the level of RN staffing and in-hospital mortality among craniotomy patients.

### Aim

The aim of this study was to investigate the relationships between RN staffing levels (in general wards, ICUs, and hospitals overall) and craniotomy inpatient mortality using a large national database of Korean National Health Insurance data, controlling for patient and hospital characteristics.

## Methods

### Design

This was a cross-sectional study to investigate the relationship between RN staffing levels and craniotomy inpatient mortality using national big data provided by the Korean National Health Insurance Service.

### Data collection

**Fig. 1:**
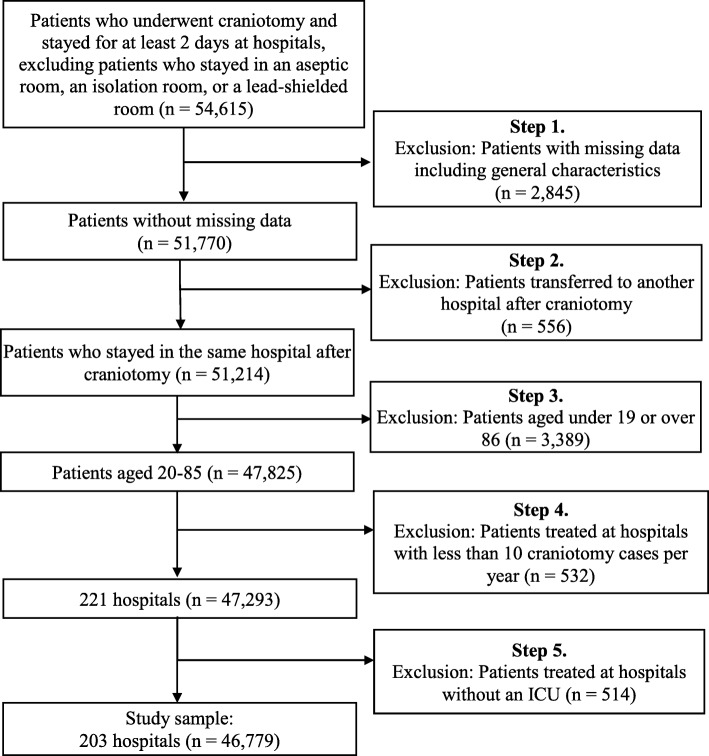
Criteria and process of subject selection

We used a large dataset provided by the National Health Insurance Service (NHIS) that contained hospital information and data on inpatient mortality after craniotomy from January 1, 2014 to December 31, 2015. This national dataset covered all hospitals and encompassed National Health Insurance members and Medical Aid recipients in Korea. To investigate the relationship between RN staffing levels and in-hospital mortality, we utilized the conceptual framework proposed by Kane, Shamliyan, Mueller, Duval, & Wilt (2007a), as well as the variables that Foster developed and the UK Department of Health used for calculating the hospital-standardized mortality ratio [21]. Then we selected the analytic variables available in the data provided by the National Health Insurance Service. The criteria and process for selecting the subjects are shown in Fig. 1. Data were gathered for subjects assigned the Korean Diagnosis-Related Group (KDRG) codes corresponding to major craniotomy except for trauma (B011-B018), other craniotomy except for trauma (cranial nerve procedure, burr hole, or trephination, B021-B022), and craniotomy for trauma treatment (B030) after undergoing craniotomy and staying for longer than 2 days at tertiary or secondary hospitals. Subjects were excluded if they were under 19 or over 86 years old [3], injured by a third party, received civil care service or palliative care, or stayed in an aseptic room, an isolation room, or a lead-shielded room. Furthermore, we collected cases of death during hospitalization for which the discharge date from the hospital in the health insurance claim data was the same as the date of death in the National Death Database administered by Statistics Korea. Finally, 46,779 subjects from 203 hospitals were included in the analysis. Our sample included 43 out of the total of 43 tertiary hospitals, 153 out of the total of 294 general hospitals, and 7 out of the total of 2868 other smaller hospitals in Korea.

### Research variables

The variables were classified into two levels: those related to hospitals and those related to patients. Hospitals were classified by type, ownership, location, number of craniotomy patients, number of beds per physician, the RN staffing level of general wards, the RN staffing level of the ICUs, and the RN staffing level of the hospitals overall. As the RN staffing level is classified using grades based on the bed-to-RN ratio in Korea, we presented the RN staffing level as the bed-to-RN ratio and grade in this study. This nursing grade system only considers the number of RNs who participate directly in patient care, not nursing assistants.

#### The registered nurse staffing level of general wards

In Korea, the nursing grade system of general wards divides hospitals into two tiers. The higher tier contains tertiary hospitals, and the lower tier includes general hospitals and other smaller hospitals. Tertiary hospitals are divided into grades 1–6: grade 1 (with a bed-to-RN ratio of less than 2.0), grade 2 (2.0–2.4), grade 3 (2.5–2.9), grade 4 (3.0–3.4), grade 5 (3.5–3.9), and grade 6 (4.0 or more). General and smaller hospitals are classified into grades 1–7: grade 1 (with a bed-to-RN ratio of less than 2.5), grade 2 (2.5–2.9), grade 3 (3.0–3.4), grade 4 (3.5–3.9), grade 5 (4.0–4.4), grade 6 (4.5–5.9), and grade 7 (6.0 or more). We adjusted this two-tier nursing grade standard by adopting the lower tier of nursing grades and adding the upper-tier grade 1 (for tertiary hospitals) as grade 0. Therefore, the RN staffing levels of general wards were re-classified into eight grades, ranging from adjusted grade 0 (with a bed-to-RN ratio of less than 2.0) to adjusted grade 7 (with a bed-to-RN ratio of 6.0 or more). To facilitate more informative comparisons, we finally categorized the RN staffing levels of general wards by combining pairs of grades, as follows bed-to-RN ratio (grade): less than 2.5:1 (0–1), from 2.5:1 to 3.4:1 (2–3), from 3.5:1 to 4.4 (4–5), and 4.5:1 or more (6–7).

#### The registered nurse staffing level of ICUs

In Korea, the RN staffing levels of ICUs are divided into grades 1–9: grade 1 (with a bed-to-RN ratio of less than 0.5), grade 2 (0.5–0.62), grade 3 (0.63–0.76), grade 4 (0.77–0.87), grade 5 (0.88–0.99), grade 6 (1.0–1.24), grade 7 (1.25–1.54), grade 8 (1.55–1.99), grade 9 (2.0 or more). We categorized the RN staffing levels of ICUs by combining pairs of grades, as follows bed-to-RN ratio (grade): less than 0.63:1 (1–2), from 0.63:1 to 0.87:1 (3–4), from 0.88:1 to 1.24:1 (5–6), and 1.25:1 or more (7–9).

#### Overall registered nurse staffing level of the hospitals

The overall bed-to-RN ratio of the hospitals was calculated as the number of total beds divided by the total number of RNs, including not only bedside RNs, but also other RNs performing administrative tasks, insurance screening, or outpatient care. In this study, the bed-to-RN ratio was classified as follows: less than 1.25:1, from 1.25:1 to 1.66:1, from 1.67:1 to 2.49:1, and 2.50:1 or more.

The patient-related factors included sex, age, type of craniotomy, severity of disease, admission route, and year of operation. The type of craniotomy was determined based on the first four digits of the KDRG. Disease severity was classified as no complications or comorbidities (CCs), minor CCs, and moderate CCs based on the sixth digit of the KDRG.

In-hospital mortality, a dependent variable, referred to death during hospitalization in cases where patients who had undergone craniotomy at a certain hospital died during the period of hospitalization at that hospital after surgery.

### Data analysis

Differences in the distribution of deaths during hospitalization depending on the characteristics of the hospitals and the patients were analyzed using the chi-square (χ^2^) test. Generalized estimating equations (GEEs) were used to characterize the relationships between RN staffing levels and mortality after craniotomy with consideration of the clustered data of subjects across hospitals. Patient outcomes, classified as either inpatient death or survival, were analyzed using GEE-based multiple logistic regression. In this study, the robust Huber-White sandwich method, which is used when there are more than 40 clusters in GEE regression, was applied [11].

## Results

### General characteristics of hospitals and craniotomy patients

Data of 29,424 patients from 43 tertiary hospitals and 17,355 patients from 160 secondary hospitals were analyzed. The distribution of hospitals according to RN staffing levels was as follows. For the general wards, 108 hospitals (53.2%) had grade 2–3 staffing (a ratio of 2.5:1–3.4:1), followed by 37 (18.2%) with the lowest level of staffing (grades 6–7, a ratio of 4.5:1 or more). The number of patients was 21,821 (46.7%) in grade 0–1 hospitals (a ratio of less than 2.5:1) and 22,517 (48.1%) in grade 2–3 hospitals (a ratio of 2.5:1–3.4:1), with approximately 95% of patients treated at hospitals with higher RN staffing levels (a ratio of less than 3.5:1). In terms of the ICU RN staffing grades, 64 hospitals (31.5%) had grade 3–4 staffing (a ratio of 0.63:1–0.87:1). There were 29,408 patients (62.9%) in hospitals with grade 1–2 staffing (a ratio of less than 0.63). In terms of the overall RN staffing grade, 24,687 patients (52.8%) were treated at hospitals with the highest RN staffing level (a ratio of less than 1.25:1) (Table 1).

**Table 1:** Characteristics of hospitals and craniotomy patients

Variables		Categories	Hospitals (*n* = 203)	Craniotomy patients (*n* = 46,779)
			n (%)	n (%)
Type		Tertiary hospital	43(21.2)	29,424(62.9)
		General hospital	160(78.8)	17,355(37.1)
Ownership		Public sector	24(11.8)	8013(17.1)
		Educational foundation	59(29.1)	21,394(45.7)
		Medical or other corporation	93(45.8)	15,107(32.3)
		Private corporation	27(13.3)	2265(4.8)
Location		Capital (Seoul)	43(21.2)	18,362(39.3)
		Metropolitan cities	54(26.6)	12,929(27.6)
		Other cities and rural areas	106(52.2)	15,488(33.1)
Number of craniotomy cases per year		10–49	99(48.8)	2833(6.1)
		50–99	41(20.2)	6069(13.0)
		100–499	41(20.2)	14,478(31.0)
		500	22(10.8)	23,399(50.0)
Number of beds per physician		< 2	14(6.9)	15,168(32.4)
		2 ≤ and < 4	46(22.7)	17,639(37.7)
		4 ≤ and < 6	55(27.1)	8991(19.2)
		≥ 6	88(43.4)	4981(10.7)
Registered Nurse (RN) staffing level	Bed-to-RN ratio in general wards (grade)	< 2.5 (0–1)	29(14.3)	21,821(46.7)
		2.5 ≤ and < 3.5 (2–3)	108(53.2)	22,517(48.1)
		3.5 ≤ and < 4.5 (4–5)	29(14.3)	783(1.7)
		≥4.5 (6–7)	37(18.2)	1658(3.5)
	Bed-to-RN ratio in the ICUs (grade)	< 0.63 (1–2)	48(23.7)	29,408(62.9)
		0.63 ≤ and < 0.88 (3–4)	64(31.5)	12,023(25.7)
		0.88 ≤ and < 1.25 (5–6)	53(26.1)	3879(8.3)
		≥1.25 (7–9)	38(18.7)	1469(3.1)
	Bed-to-RN ratio in the hospitals overall	< 1.25	43(21.2)	24,687(52.8)
		1.25 ≤ and < 1.67	54(26.6)	13,920(29.8)
		1.67 ≤ and < 2.50	54(26.6)	4764(10.2)
		≥ 2.50	52(25.6)	3408(7.3)

### Mortality after craniotomy by characteristics of hospitals and patients

Among the 46,779 patients who received craniotomy, 3315 died during hospitalization, yielding a mortality rate of 7.1%. The mortality rate was significantly different according to all characteristics of the hospitals (Table 2). The mortality rate of patients after craniotomy increased with higher bed-to-RN ratio. As the bed-to-RN ratio increased, mortality increased. The lowest mortality rate (4.0%) was found at hospitals with a bed-to-RN ratio of less than 2.5:1 in the general wards. The mortality rate of hospitals with more than 4.5 beds per RN was 19.5%, which was 4.9 times higher than the rate in hospitals with less than 2.5 beds per RN (χ^2^ = 948.91, *p* < .001). As the bed-to-RN ratio in the ICU increased, the mortality rate also increased (χ^2^ = 949.14, *p* < .001), and a similar trend was found for mortality rates to be higher at hospitals with a higher overall bed-to-RN ratio (χ^2^ = 523.71, *p* < .001).

**Table 2:** Mortality of craniotomy patients by characteristics of hospitals

Variables		Categories	Survival (*n* = 43,464)	Death (*n* = 3315)	χ ^2^ (*p*)
			n (%)	n (%)	
Type		Tertiary hospital	27,977(95.1)	1447(4.9)	566.55 (<.001)
		Secondary hospital	15,487(89.2)	1868(10.8)	
Ownership		Public sector	7700(96.1)	313(3.9)	179.86 (<.001)
		Educational foundation	19,661(91.9)	1733(8.1)	
		Medical or other corporation	14,057(93.1)	1050(7.0)	
		Private corporation	2046(90.3)	219(9.7)	
Location		Capital (Seoul)	17,626(96.0)	736(4.0)	441.85 (<.001)
		Metropolitan cities	11,812(91.4)	1117(8.6)	
		Other cities and rural areas	14,026(90.6)	1462(9.4)	
Number of craniotomy patients		10–49	2391(84.4)	442(15.6)	1232.37 (<.001)
		50–99	5267(86.8)	802(13.2)	
		100–499	13,187(91.1)	1291(8.9)	
		500	22,619(96.7)	780(3.3)	
Number of beds per physician		< 2	14,823(97.7)	345(2.3)	950.40 (<.001)
		2 ≤ and < 4	16,202(91.9)	1437(8.2)	
		4 ≤ and < 6	8123(90.4)	868(9.7)	
		≥ 6	4316(86.7)	665(13.4)	
Registered Nurse (RN) staffing level	Bed-to-RN ratio in general wards (grade)	< 2.5 (0–1)	20,946(96.0)	875(4.0)	948.91 (<.001)
		2.5 ≤ and < 3.5 (2–3)	20,544(91.2)	1973(8.8)	
		3.5 ≤ and < 4.5 (4–5)	640(81.7)	143(18.3)	
		≥4.5 (6–7)	1334(80.5)	324(19.5)	
	Bed-to-RN ratio in the ICUs (grade)	< 0.63 (1–2)	28,107(95.6)	1301(4.4)	949.14 (<.001)
		0.63 ≤ and < 0.88 (3–4)	10,724(89.2)	1299(10.8)	
		0.88 ≤ and < 1.25 (5–6)	3425(88.3)	454(11.7)	
		≥1.25 (7–9)	1208(82.2)	261(17.8)	
	Bed-to-RN ratio in the hospitals overall	< 1.25	23,461(95.0)	1226(5.0)	523.71 (<.001)
		1.25 ≤ and < 1.67	12,801(92.0)	1119(8.0)	
		1.67 ≤ and < 2.50	4281(89.9)	483(10.1)	
		≥2.50	2921(85.7)	487(14.3)	

### Mortality after craniotomy by characteristics of patients

The mortality rate of patients after craniotomy differed by sex, age, type of craniotomy, degree of complications and comorbidities (CCs), and admission route (Table 3). The mortality rate after craniotomy for male subjects was significantly higher than that for female subjects (χ^2^ = 126.40, *p* < .001), and mortality was more common in older patients (χ^2^ = 130.98, *p* < .001).

**Table 3:** In-hospital mortality of craniotomy patients by characteristics of patients

Variables	Categories	Survival (*n* = 43,464)	Death (*n* = 3315)	χ ^2^ (*p*)
		n (%)	n (%)	
Sex	Male	21,821(91.6)	2000(8.4)	126.40 (<.001)
	Female	21,643(94.3)	1315(5.7)	
Age (year)	20–29	1441(96.5)	53(3.6)	130.98 (<.001)
	30–39	2954(95.2)	149(4.8)	
	40–49	7155(93.9)	464(6.1)	
	50–59	11,658(93.3)	841(6.7)	
	60–69	9885(92.7)	783(7.3)	
	≥70	10,371(91.0)	1025(9.0)	
Type of craniotomy	Major craniotomy (excluding trauma)	28,540(95.5)	1353(4.5)	858.01 (<.001)
	Other craniotomy (excluding trauma)	6335(87.1)	941(12.9)	
	Craniotomy for trauma	8589(89.4)	1021(10.6)	
Complications and comorbidities	None	24,527(97.3)	679(2.7)	2454.81 (<.001)
	Minor	11,860(92.0)	1036(8.0)	
	Moderate	7077(81.6)	1600(18.4)	
Admission route	Outpatient department	26,662(97.7)	622(2.3)	2297.39 (<.001)
	Emergency department	16,802(86.2)	2693(13.8)	
Year of operation	2014	21,760(92.7)	1712(7.3)	3.07 (.080)
	2015	21,704(93.1)	1603(6.9)	

The mortality rate was 12.9% in the category of other craniotomy (χ^2^ = 858.01, *p* < .001) and 18.4% in the moderate CCs group (χ^2^ = 2454.81, *p* < .001). The mortality rate of patients admitted through the emergency department was significantly higher (13.8%) than that of patients admitted through the outpatient department (2.3%) (χ^2^ = 2297.39, *p* < .001).

### Influence of the level of registered nurse staffing on inpatient mortality

To control for all variables, including hospital-related factors and patient-related factors, that could have affected inpatient mortality after craniotomy, those variables were entered into the regression equations along with the level of RN staffing to determine the net effects of the RN staffing level on in-hospital mortality after craniotomy. Three models were constructed: model 1 (RN staffing level in the general ward), model 2 (RN staffing level in the ICU), and model 3 (overall RN staffing level of the hospital).

The results are shown in Table 4. The RN staffing level had a consistent effect on in-hospital mortality in model 1, model 2, and model 3. In model 1, after controlling for all other factors, the mortality rate was 51% lower in hospitals with a bed-to-RN ratio of less than 2.5:1 (95% CI = 0.29–0.85), and 50% lower in hospitals with a ratio of 2.5:1–3.4:1 (95% CI = 0.33–0.78) than in hospitals with a ratio of 4.5:1 or more. In model 2, the mortality rate was 56% lower in hospitals with a ratio of less than 0.63:1 in the ICU (95% CI = 0.27–0.70) and 50% lower in hospitals with a ratio of 0.63:1–0.87:1 (95% CI = 0.33–0.76) than in hospitals with a ratio of 1.25:1 or more. In model 3, the odds ratios (ORs) for mortality of patients in hospitals with ratios of less than 1.25:1 and 1.25:1–1.66:1 were 0.64 (95% CI = 0.46–0.89) and 0.70 (95% CI = 0.51–0.94), respectively, compared to hospitals with a ratio of 2.5:1 or more, reflecting a significant discrepancy. The RN staffing level had the strongest effect on mortality of all the analyzed variables.

**Table 4:** GEE logistic regression for inpatient mortality of craniotomy patients by registered nurse staffing level (*N* = 46,779)

Factors	Variables	Categories	Model 1			Model 2			Model 3		
			OR	95% CI	*p*	OR	95% CI	*p*	OR	95% CI	*p*
Patient	Age (year)		1.01	(1.01–1.01)	<.001	1.01	(1.01–1.01)	<.001	1.01	(1.00–1.01)	<..001
	Sex (ref = female)	Male	1.06	(0.99–1.14)	.112	1.07	(0.99–1.15)	.090	1.06	(0.98–1.14)	.146
	Type of craniotomy (ref = craniotomy for trauma)	Major craniotomy	1.10	(0.98–1.24)	.116	1.10	(0.97–1.24)	.125	1.11	(0.98–1.26)	.109
		Other craniotomy	1.74	(1.52–2.00)	<.001	1.74	(1.52–2.00)	<.001	1.69	(1.48–1.94)	<.001
	Complications and comorbidities (ref = moderate)	No	0.29	(0.23–0.37)	<.001	0.30	(0.23–0.38)	<.001	0.28	(0.22–0.36)	<.001
		Minor	0.50	(0.45–0.56)	<.001	0.50	(0.45–0.56)	<.001	0.49	(0.44–0.55)	<.001
	Admission route (ref = outpatient)	Emergency department	4.43	(3.57–5.50)	<.001	4.65	(3.77–5.74)	<.001	4.37	(3.51–5.45)	<.001
	Year of operation (ref = 2014)	2015	0.91	(0.84–0.98)	.019	0.91	(0.84–0.98)	.019	0.91	(0.84–0.98)	.018
Hospital	Type (ref = Secondary hospital)	Tertiary hospital	1.14	(0.90–1.44)	.268	1.16	(0.95–1.42)	.149	1.22	(0.98–1.52)	.076
	Ownership (ref = private corporation)	Public sector	0.76	(0.45–1.26)	.287	0.88	(0.51–1.50)	.632	0.75	(0.43–1.30)	.304
		Educational foundation	1.21	(0.75–1.96)	.431	1.40	(0.85–2.30)	.190	1.26	(0.76–2.10)	.367
		Medical or other corporation	1.04	(0.66–1.64)	.859	1.16	(0.73–1.85)	.525	1.05	(0.66–1.67)	.846
	Location (ref = other cities and rural areas)	Capital	1.09	(0.87–1.36)	.440	1.19	(0.96–1.47)	.115	1.09	(0.90–1.33)	.380
		Metropolitan cities	1.20	(0.98–1.47)	.082	1.27	(1.04–1.55)	.019	1.19	(0.97–1.46)	.094
	Number of craniotomy patients (10-patient increments)		0.99	(0.99–1.00)	<.001	0.99	(0.99–1.00)	<.001	0.99	(0.99–0.99)	<.001
	Number of beds per physician		1.02	(0.99–1.05)	.192	1.03	(1.00–1.06)	.096	1.04	(1.01–1.07)	.002
Registered nurse (RN) staffing level	Bed-to-RN ratio in general wards (ref = 4.5 or more (6–7))	< 2.5 (0–1)	0.49	(0.29–0.85)	.010						
		2.5 ≤ and < 3.5 (2–3)	0.50	(0.33–0.78)	.002						
		3.5 ≤ and < 4.5 (4–5)	0.88	(0.54–1.42)	.592						
	Bed-to-RN ratio in the ICUs (ref = 1.25 or more (7–9))	< 0.63 (1–2)				0.44	(0.27–0.70)	.001			
		0.63 ≤ and < 0.88 (3–4)				0.50	(0.33–0.76)	.001			
		0.88 ≤ and < 1.25 (5–6)				0.83	(0.57–1.19)	.311			
	Bed-to-RN ratio in the hospitals overall (ref = 2.50 or more)	< 1.25							0.64	(0.46–0.89)	.007
		1.25 ≤ and < 1.67							0.70	(0.51–0.94)	.020
		1.67 ≤ and < 2.50							0.78	(0.57–1.06)	.111

*OR* Odds ratio, *CI* Confidence interval

In addition to the RN staffing level, patient-level variables (age, craniotomy type, CCs, and admission route) and a hospital-level variable (number of craniotomy patients) were found to have a statistically significant effect on in-hospital mortality after craniotomy in all three models. At the patient level, the mortality rate of craniotomy patients increased by 1% for each additional year of age. Mortality was significantly lower among patients with no or minor CCs. The OR for mortality was significantly higher among patients admitted via the emergency department than among those admitted from an outpatient department. In terms of craniotomy type, the category of other craniotomy was associated with a significantly higher mortality rate than was observed in patients who underwent craniotomy due to trauma.

At the hospital level, mortality decreased by 1% for each increment of 10 craniotomy patients. No significant effect was found according to hospital type and ownership. Only model 3 showed a statistically significant effect of the number of beds per physician on mortality. The mortality rate of craniotomy patients increased by 4% for each additional bed per physician (95% CI = 1.01–1.07).

## Discussion

In this study using a large national database, the mortality was significantly different according to all characteristics at the patient and hospital level. We assessed RN staffing levels in terms of the bed-to-RN ratio in general wards, ICUs, and the hospitals overall, controlling for the characteristics of the patients and hospitals, in order to identify the impacts of RN staffing levels on the in-hospital mortality rate after craniotomy. Among the hospital-related factors that could affect in-hospital mortality after craniotomy, the RN staffing level had the greatest impact. We found threshold levels of RN staffing that meaningfully impacted the inpatient mortality rate. First, the probability of death in craniotomy patients who received care at hospitals with a bed-to-RN ratio of less than 3.5:1 in general wards was more than 50% lower than those who received care at hospitals with a bed-to-RN ratio of 4.5:1 or more, respectively. This results are similar to those of previous research finding lower levels of RN staffing to be associated with a higher likelihood of death in surgical patients, with risk elevations of 57, 78, and 199% in hospitals with RN staffing grade 2–3, grade 4–5, and grade 6–7, respectively, than in those with grade 0–1 [11]. Our results also show that the probability of death among patients in surgery wards with less than two patients per shift RN was 38% lower than that among patients with more than five patients per shift RN [22], and that lower RN staffing levels were associated with higher inpatient mortality rates among surgical patients [1, 9, 23, 24]. In addition, the in-hospital mortality rates of craniotomy patients at hospitals with a ratio of 3.5:1 or more were not significantly different in this study. Therefore, we suggest that the level of RN staffing for craniotomy patients should be improved to a bed-to-RN ratio of less than 3.5:1 in general wards, as a minimum standard, to reduce the in-hospital mortality rate after craniotomy.

Second, our results can be interpreted as suggesting that the risk of in-hospital mortality of craniotomy patients could be reduced by more than 50% by lowering the bed-to-RN ratio in the ICUs from 1.25:1 to less than 0.88:1. Additionally, there was no significant difference in the mortality rate of craniotomy patients at hospitals with a ratio of 0.88:1 or more in the ICUs. This results are consistent with the previous finding that the mortality rate of patients increased by 9% (i.e., 15 more deaths per 1000 patients) when ICU RNs had to take care of an additional patient at secondary hospitals [9], and heavier workloads of ICU RNs were associated with lower survival rates of critical patients in the ICU [25]. Therefore, we suggest that the bed-to-RN ratio should be less than 0.88:1 in ICUs to reduce the mortality rate after craniotomy.

Third, the risk of in-hospital mortality among craniotomy patients could be reduced by more than 30% by reducing the overall hospital bed-to-RN ratio from 2.5:1 or more to less than 1.67:1. Cho et al. (2015) found that the patient mortality rate increased by 5% for each extra patient added to the RN’s workload [10]. Ball et al. (2018) argued that missed care acted as the mediating variable in the mechanism through which the RN staffing level affected the mortality rate of surgical patients [26]. The results of this study support those of previous studies arguing that appropriate RN staffing levels improved nursing quality and decreased patient mortality [3, 7–11, 13, 26, 27]. Thus, we suggest that the bed-to-RN ratio should be less than 1.67:1 in overall hospitals.

However, the administrators of hospitals tend to reduce nursing staff to minimize costs [28]. Health care personnel account for more than 50% of hospital costs, of which RNs comprise the largest proportion. Hospital administrators and funders believe that simply reducing the number of RNs will decrease hospital costs, so reducing the number of RNs is often their first attempt when embarking upon cost-cutting initiatives. RNs should show that they are cost-effective health care personnel, but it is not easy to demonstrate the cost-effectiveness of nursing staff. However, it is clear that nursing staff have a beneficial effect on patient outcomes [29]. The association of higher levels of RN staffing with lower in-hospital mortality rates could be explained by the possibility that an appropriate level of RN staffing could prevent RNs from making mistakes or missing changes in patients’ condition due to a heavy workload [11]. In actual patient care, although RNs provide team-based nursing care with diverse medical staff and assistants, some countries recognize the importance of RNs and set mandatory standards for the number of RNs. In California, USA, the patient-to-RN staffing standard is 2:1 in ICUs and 5:1 in general medical and surgical wards. In Victoria, Australia, it is 2–3:1 in ICUs and 4–6:1 in general wards [30]. The importance of adequate levels of RN staffing is increasingly recognized, as mandated RN staffing standards (patient-to-RN ratios) are enacted and enforced in many countries [31]. Therefore, it is necessary to establish standards of RN staffing levels and to impose penalties for violating those staffing levels by referring to standard RN staffing levels.

In Korea, based on a 40-h work week, RNs work 230 days per year in the wards, with the exclusion of vacation and training days [4]. Considering the 90% bed occupancy rate and the three-shift duty schedule, the results of this study suggest that each RN should be in charge of 15.1 patients in general wards and 3.8 patients in the ICUs. This is not a strict criterion in the international context. Therefore, in order to reduce mortality in craniotomy patients, who have the highest postoperative mortality rate, the findings from our study suggest that the bed-to-RN ratio should be less than 3.5:1 in general wards and less than 0.88:1 in ICUs. It is also important to note that the criterion of a ratio of 2.5:1 or more for overall hospitals used in this study is similar to that of the criterion of 2.5 inpatients per RN used to estimate the number of RNs under the Korean Enforcement Rule of the Medical Service Act. Our finding that the mortality rate decreased when the bed-to-RN ratio was less than 1.67:1 suggests that the bed-to-RN ratio should be increased in Korea from the current 2.5:1 to 1.66:1. In this context, the findings of this study could furnish useful criteria for calculating the adequate RN staffing level. It is necessary to develop a scientific basis for mandating appropriate RN staffing standards to guarantee the people’s health rights and to ensure patient safety.

### Limitations

Our study has several limitations. First, we analyzed the bed-to-RN ratio per quarter, a standard method of classifying the level of RN staffing in Korea that does not reflect the actual nursing workload and mortality per shift per day. Second, the number of beds is not the same as the number of patients. Since the bed occupancy rate is not 100%, the actual number of patients may be less than the number of beds. Third, we only analyzed the number of RNs and physicians since data were not available on other medical staff and nursing assistants. Furthermore, we did not evaluate the competence of RNs, which influences the quality and results of patient care. Finally, this study has limitations in the degree to which it can be used to infer causal relationships between RN staffing levels and in-hospital mortality in craniotomy patients, since it was a cross-sectional study.

## Conclusions

This study analyzed the net effects of the level of RN staffing by controlling for the characteristics of patients and hospitals with regard to mortality in craniotomy patients. We believe that this study is meaningful, as it is the first study to analyze the effects of RN staffing levels on the in-hospital mortality rate of craniotomy patients using a large national dataset covered all hospitals in Korea. The results provide evidence that higher RN staffing level was associated with a lower risk of in-hospital mortality of craniotomy patients in general wards, ICUs, and hospitals overall. Thus, a sufficient staffing level of RNs should be ensured to reduce in-hospital mortality. In the future, we suggest that a longitudinal study should include variables such as the RN staffing level in a way that takes into account the actual nursing workload, the actual number of patients, and the number of diverse medical staff and assistants. In addition, future research should also include variables to assess the competence and training of RNs working with patients with high levels of need.

## Data Availability

The data that support the findings of this study are available from the National Health Insurance Service (NHIS) but restrictions apply to the availability of these data, which were used under license for the current study, and so are not publicly available. Data are however available from the authors upon reasonable request and with permission of the NHIS.

## Abbreviations

CCs: Complications and comorbidities
CI: Confidence interval
GEE: Generalized estimating equation
ICU: Intensive care unit
KDRG: Korean Diagnosis-Related Group
NHIS: National Health Insurance Service
OR: Odds ratio
RN: Registered nurse
